# Correlation and Influencing Factors Between Laryngopharyngeal Reflux Disease and Sleep Status in Patients

**DOI:** 10.3389/fsurg.2022.845653

**Published:** 2022-02-09

**Authors:** Yue Liu, Jian Wu, Feng Xiao, Xiaofeng Gu, Li Ji

**Affiliations:** Department of Otolaryngology, The Second People's Hospital of Changzhou, Changzhou, China

**Keywords:** laryngopharyngeal reflux disease, sleep disorders, reflux symptom index, influencing factor, depressive mood

## Abstract

**Objective:**

To observe the correlation between laryngopharyngeal reflux disease (LPRD) and patients' sleep status, and to explore the related factors of LPRD.

**Methods:**

Four hundred and sixteen patients who visited the otorhinolaryngology clinic in our hospital from June 2019 to June 2021 were selected as the research subjects. According to the scale of reflux symptom index, the subjects were divided into a patients group (120 patients) with an the reflux symptom index (RSI) > 13 and a control group (296 patients) with an RSI ≤ 13 according to the RSI scale score. General patient information was collected. The sleep state and emotional state of patients in the two groups were evaluated, and the related influencing factors for LPRD were also evaluated. The correlation between sleep state and depression in LPRD patients was analyzed.

**Results:**

Four hundred and sixteen patients were divided into patients group and control group according to RSI score, the ratio of the two groups was 1:2.47. In the patients group, the common symptoms of RSI score and the top three of the total score were as follows: Foreign body sensation in throat in 112 patients, 438 points; Keep voice clear in 108 patients, 381 points; Excessive phlegm or nasal discharge reflux in 101 patients, 348 points. The PSQI and HADS scores in the patients group were higher than those in the control group (*t* = 19.990, 13.007, 14.690, *P* all <0.001). Logistic regression analysis showed that high-fat diet and high PSQI score were the risk factors for the development of LPRD (*P* = 0.012, *P* = 0.007). According to the PSQI score, the patients in the patients group were divided into 35 patients with abnormal PSQI score, 85 patients with normal PSQI score, and the HADS scores of those with abnormal PSQI score were all lower than those with normal PSQI score (*P* > 0.05). The PSQI score of the patients in the patients group was positively correlated with the HADS score (*r* = 0.714, *P* = 0.013).

**Conclusion:**

Sleep disorder may lead to the occurrence or aggravation of anxiety and depression in patients with LPRD, and it is an independent risk factor for the development of LPRD. Clinical attention to the treatment of sleep disorders in patients with LPRD may be conducive to improving the efficacy of LPRD.

## Introduction

Reflux diseases include gastroesophageal reflux disease and laryngopharyngeal reflux disease (LPRD), in which LPRD is a clinical syndrome caused by reflux of stomach contents to the upper esophageal sphincter. Clinical manifestations include foreign body sensation in the throat, pain, persistent throat clearing, hoarseness, chronic cough, asthma, and so on. If it is not effectively controlled in time, it can cause serious problems such as laryngeal disappearance, granuloma, subglottic stenosis, and so on, which will seriously affecting the daily life and work quality of patients (1–3). Reflux diseases such as LPRD disease are caused by various pathophysiological abnormalities, such as dynamic changes, increased visceral sensitivity, and disturbance of brain-gut axis regulation. Some psychological factors, such as life stress, social stress, and psychological state, are caused by the interaction of the central nervous system and intestinal nervous system of the brain (4, 5).

The earliest objective reflection of human body's response to stress events is sleep behavior disorder. Studies have pointed out that sleep disorders is closely related to the occurrence of intestinal symptoms, and patients with reflux disease are often accompanied by sleep quality decline or sleep disorders (6). Previous reports paid more attention to the effects of smoking and drinking on the LPRD, but there was little research on the sleep state of this group. In recent years, with the change of modern lifestyle, people's circadian rhythm has been disrupted, and sleep deprivation has become an increasingly common problem. The proportion of normal people, especially young people, with unhealthy living habits such as sleep disorder and high-fat diet has increased significantly. At the same time, studies have confirmed that patients with LPRD disease are usually accompanied by psychological disorders, and anxiety and depression are more common in people with sleep disorders (7). In this study, we conducted a comprehensive analysis of the clinical symptoms, sleep, and diet of the patients seeking treatment, aiming to explore the correlation between LPRD and the sleep status of patients and the influencing factors of their onset, so as to provide more data reference for the clinical prevention and treatment of LPRD.

## Data and Methods

### General Information

Total of 416 patients who visited the otorhinolaryngology outpatient department from June 2019 to June 2021 were selected as the research subjects. Inclusion criteria of patients: age ≥ 18 years; Patients cooperated with the research and signed informed consent form; Clinical data are complete. Patient exclusion criteria: malignant tumor; Heart disease; Hypertension; Diabetes; Take anti-acid drugs for nearly 2 weeks. The subjects were divided into a patients group (120 patients) with > 13 points and a control group (296 patients) with ≤ 13 points according to reflux symptom index (RSI) scale score. This study was approved by the Medical Ethics Committee of our hospital, with the informed consent of the patients.

### Research Methods

General patient information was collected, including gender, age, height, weight, and medical history. The medical history mainly includes whether there are stomach diseases such as gastric ulcer and chronic gastritis. Have a history of catching cold; Whether there is too much work and study pressure; Anxiety and depression, etc. Patients were evaluated for smoking, alcohol consumption, constipation, and dietary habits including a high-fat diet. The smoking criterion is to smoke more than one cigarette every day for 6 months continuously or cumulatively. The criterion of drinking is drinking for 3 days or more per week, 25 grams or more for men, and 15 grams or more for women. The criterion of constipation is difficulty and hard defecation 3 months before admission; There is defecation but can't be discharged, defecation frequency is reduced or defecation is not complete, defecation is less than 3 times a week, defecation weight is less than 35 g/d, and defecation is laborious more than 25% of the time. The judging standard of high-fat diet is that fat > 50 g/d and fish protein > 225 g/d are carried out three or more days a week.

The Pittsburgh sleep quality index (PSQI) scale was used to assess the sleep status of the two groups. PSQI > 7 points indicated the existence of sleep disorders, and PSQI ≤ 7 points indicated normal. The hospital anxiety and depression scale (HADS) was used to assess the emotional state of the two groups. PSQI > 7 points indicated that there might be depression and anxiety, and HADS ≤ 7 indicated that there was no depression and anxiety problem.

In order to analyze the correlation between sleep status and depression in patients with LPRD, and then to analyze the related influencing factors of LPRD, the differences of PSQI and HADS scores between the two groups were compared.

### Statistical Methods

SPSS22.0 software was used for processing. The measurement data of the experimental data were expressed as mean standard deviation (x ± s), and *t*-test was used for pairwise comparison. The enumeration data were expressed as (%) and the comparison was conducted by χ^2^ test. Multivariate Logistic regression analysis was used for multivariate analysis. Pearson correlation was used for correlation analysis between scores. The test level was α = 0.05, and *P* < 0.05 indicated that the difference was statistically significant.

## Results

### Study Subject Composition and RSI Score

Among the 416 patients, there were 244 males and 172 were females, with the ratio of male to female being 1.42:1, RSI 0–6 score of 83 patients, 7–13 score of 37 patients and >13 score of 296 patients. According to RSI score, the patients were divided into two groups: the patients group (*n* = 120) and control group (*n* = 296), with the ratio of 120:296 = 1:2.47. In the patients group, the common symptoms of RSI score and the top three of the total score were as follows: Foreign body sensation in throat in 112 patients, 438 points; Keep voice clear in 108 patients, 381 points; Excessive phlegm or nasal discharge reflux in 101 patients, 348 points. See Table 1 for details.

**Table 1:** Patients and scores of reflux symptom index scale in patients group.

**Clinical symptoms**	**Number of patients**	**Percentage (%)**	**Scoring (score)**
Foreign body sensation in throat	112	93.33	438
Keep voice clear	108	90.00	381
Excessive phlegm or nasal discharge reflux	101	84.17	348
Cough	87	72.50	270
Hoarseness or dysphonia	82	68.33	233
Cough after eating or after lying down	79	65.83	217
Dyspnea or recurrent episodes of suffocation	77	64.17	185
Burning sensation in the stomach, chest pain, indigestion or stomach pain	63	52.50	160
Difficulty in swallowing food, liquids, tablets	59	49.17	157

### Comparison of General Data Between the Two Groups

The differences in alcohol, high-fat diet, and gastric disease history between the two groups were statistically significant (*P* <0.05). There was no significant difference in gender, age, BMI, occupation, smoking, constipation, catching cold and stress between the two groups (*P* > 0.05). See Table 2 for details.

**Table 2:** Comparison of general data between the two groups (*n*, %).

**Variable**		**Control group (*n* = 296)**	**Patients group (*n* = 120)**	***χ^2^ value***	***P-value***
Gender	Male	171 (57.77)	73 (60.83)	0.330	0.565
	Female	125 (42.23)	47 (39.17)		
Age (years)	>50	53 (17.91)	23 (19.17)	0.105	0.949
	31–50	127 (42.91)	50 (41.67)		
	18–30	116 (39.19)	47 (39.17)		
BMI (kg/m^2^)	≤ 24	180 (60.81)	76 (63.33)	0.230	0.632
	>24	116 (39.19)	44 (36.67)		
Occupation	Brainpower	47 (15.88)	18 (15.00)	1.260	0.533
	Physical strength	121 (40.88)	43 (35.83)		
	Freelance	128 (43.24)	59 (49.17)		
Smoke	Yes	68 (22.97)	33 (27.50)	0.952	0.329
	No	228 (77.03)	87 (72.50)		
Alcohol	Yes	51 (17.23)	37 (30.83)	9.474	0.002
	No	245 (82.77)	83 (69.17)		
Constipation	Yes	29 (9.80)	13 (10.83)	0.101	0.751
	No	267 (90.20)	107 (89.17)		
High-fat diet	Yes	91 (30.74)	49 (40.83)	3.893	0.048
	No	205 (69.26)	71 (59.17)		
Catch cold	Yes	82 (27.70)	37 (30.83)	0.410	0.522
	No	214 (72.30)	83 (69.17)		
Pressure	Yes	56 (18.92)	24 (20.00)	0.064	0.800
	No	240 (81.08)	96 (80.00)		
Gastric disease history	Yes	92 (31.08)	74 (61.67)	33.308	<0.001
	No	204 (68.92)	46 (38.33)		

### Comparison of PSQI and HADS Scores Between the Two Groups

**Figure 1:**
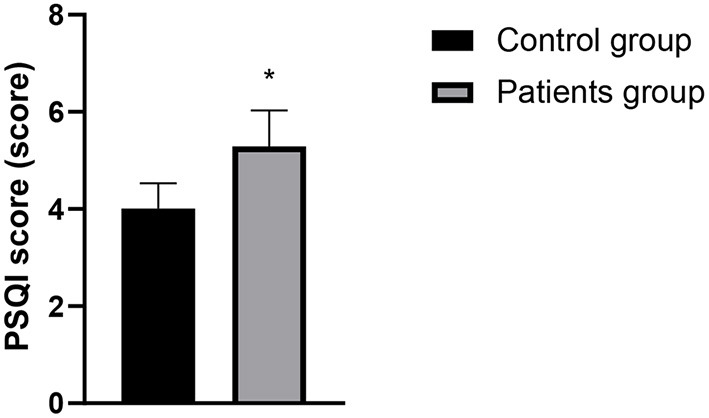
Comparison of PSQI scores between the two groups. Compared with the control group, *P < 0.05.

**Figure 2:**
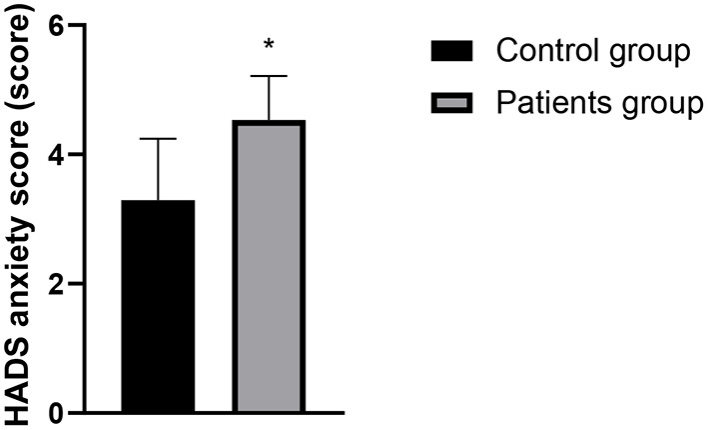
Comparison of HADS anxiety scores between the two groups. Compared with the control group, *P < 0.05.

**Figure 3:**
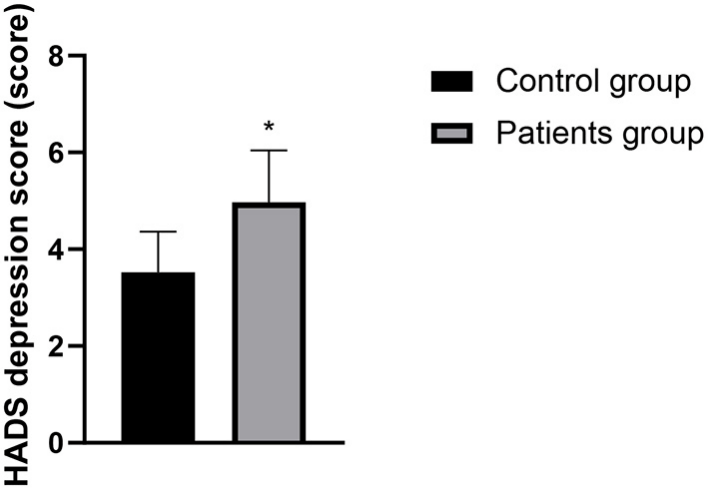
Comparison of HADS depression scores between the two groups. Compared with the control group, *P < 0.05.

The PSQI score, HADS anxiety score and HADS depression score of the patients in the case group were higher than those in the control group, and the differences were statistically significant (*P* < 0.05). See Figures 1–3 in detail.

### Analysis of Multiple Factors Affecting the Onset of LPRD

Logistic regression analysis showed that high-fat diet and high PSQI score were the risk factors for the development of LPRD (*P* < 0.05). See Tables 3, 4 for details.

**Table 3:** Assignment for multivariate analysis of factors.

**Factors**	**Variables**	**Assignment**
Alcohol	X1	No = 0, Yes = 1
High-fat diet	X2	No = 0, Yes = 1
Gastric disease history	X3	No = 0, Yes = 1
PSQI score	X4	Continuous variable
HADS score	X5	Continuous variable

**Table 4:** Analysis of multiple factors affecting the onset of LPRD.

**Factors**	***B***	***S.E***	***Walds***	***P***	***OR***	***95% CI***
Alcohol	0.463	0.294	2.480	0.113	1.589	0.893–2.827
High-fat diet	0.791	0.224	12.469	0.012	2.206	1.422–3.421
Gastric disease history	0.511	0.296	2.980	0.059	1.667	0.933–2.978
PSQI score	1.095	0.309	12.558	0.007	2.989	1.631–5.477
HADS score	0.416	0.307	1.273	0.127	1.397	0.782–2.495

### Analysis of the Correlation Between Sleep State and Depression and Anxiety in Patients of Patients Group

**Figure 4:**
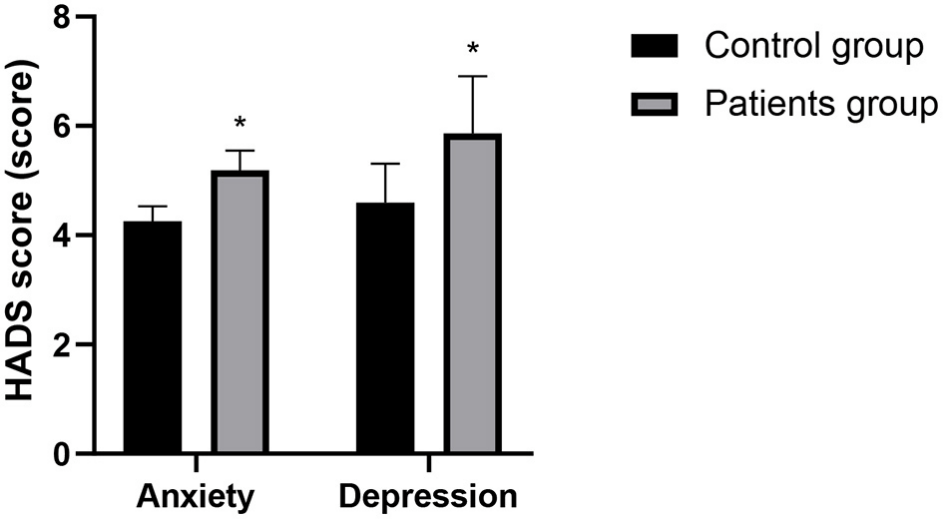
Comparison of HADS scores between the abnormal PSQI and normal PSQI. Compared with the control group, *P < 0.05.

The patients in the patients group were divided into two groups according to PSQI scores, including 35 patients with abnormal PSQI scores, 85 patients with normal PSQI scores, and the HADS anxiety score (5.19 ± 0.36) and HADS depression score (5.86 ± 1.05) of those with abnormal PSQI scores were all higher than those with normal PSQI scores (4.26 ± 0.27, 4.60 ± 0.71), with statistically significant difference (*P* < 0.05). The PSQI score of the patients in the patients group was positively correlated with the HADS score (*r* = 0.714, *P* = 0.013). See Figure 4 for details.

## Discussion

A recent multi-center epidemiological survey in China shows that the incidence of LPRD among outpatients in otolaryngology in China was about 10%, and there are regional, gender and age differences (8). LPRD can cause adult asthma, idiopathic pulmonary fibrosis and sinusitis, otitis media, tumors, and other otorhinolaryngology diseases. Therefore, it has been paid more and more attention in the clinical field, hoping to deepen the understanding of the pathophysiology of LPRD, and to find new targets for the diagnosis, prevention, and treatment of diseases (9).

Studies have found that patients with LPRD have decreased vagal tone and dysfunction, suggesting that vagal disorders are involved in the occurrence and development of LPRD (10). The vagus nerve is widely distributed in the throat, esophagus, gastrointestinal tract, lung, heart and other organs, among which throat is the most sensitive part. The abnormal vagus nerve function will cause chronic cough, foreign body sensation in the throat, frequent throat cleaning and so on (11). The results of this study showed that the top three common symptoms of RSI score and comprehensive scores in the patients group were pharyngeal foreign body sensation, continuous throat clearing, excessive sputum or nasal discharge. Reflux stimulates the cough receptors in the larynx and C fiber in the tracheal wall. The stimulation is introduced by the vagus nerve. It may interact with each other at the afferent nerves or nerves and cause cross-sensitization of esophagus and bronchi. That is to say, reflux increases the sensitivity of cough or directly leads to cough onset. However, cough once again increases the relaxation reaction of esophageal sphincter, which leads to LPRD disease (12).

There is a two-way regulatory effect between digestive function and brain in human body, that is, through the brain-gut axis and the sympathetic/parasympathetic nervous system, the central nervous system and the digestive system are closely linked to form a complete dynamic feedback loop to regulate biological rhythm and sleep state, so there is a correlation between sleep disorders and changes in digestive function (13). Changes of modern lifestyle disrupts the circadian rhythms, and sleep disorders has become an increasingly common problem. Sleep disorders include difficulty falling asleep, frequent awakening, abnormal sleep structure, short deep sleep period, fast eye movement sleep period, short total sleep time in a day, etc. The research by Pandey and Kar (14) confirmed that more than 25% of patients with gastroesophageal reflux disease have sleep disorders, and sleep disorders can also affect the development of gastroesophageal reflux disease through multiple pathways. However, there are relatively few reports on whether LPRD is related to sleep disorders. The results of this study showed that the PSQI and HADS scores of patients in the patients group were higher than those in the control group. It indicated that sleep disorder, depression, and anxiety might be closely related to the occurrence of LPRD. The clinical research by Teklu et al. (15) has confirmed that the relationship between sleep state and reflux diseases may be two way, and reflux may cause uncomfortable symptoms and may also disturb sleep state. Heartburn is also a representative symptom at night, which often causes GERD patients to wake up for a short time, and affects the overall sleep quality. Anxiety, depression and excessive pressure can cause gastric peristalsis slowdown in patients, which should be related to LPRD in theory. According to reports by Caparroz et al. (13), most patients with LPRD disease have poor autonomic nerve regulation function and increased sympathetic activity, so they often suffer from anxiety and/or depression.

The study also shown that patients in the patients group had a higher drinking history, high fat diet history and stomach diseases than those in the control group. Since the mid-1980s, there has been frequent reports of pharyngeal reflux among throat cancer patients with a history of alcohol and tobacco abuse. The incidence of gastroesophageal reflux in smoking and drinking patients is high, and it is speculated that tobacco, alcohol and gastroesophageal reflux have a synergistic effect on the etiology of pharyngeal cancer. Further logistic regression analysis showed that high-fat diet and sleep disorder were the independent risk factors for LPRD. It is pointed out that fat intake, especially cholesterol and saturated fatty acid intake, is positively correlated with the occurrence of gastroesophageal reflux disease (16). It is speculated that the pathogenesis may be related to the fact that a high-fat diet can delay gastric emptying and reduce lower esophageal sphincter pressure, making reflux more likely to occur, thus inducing cough and other LPRD symptoms.

At present, it is considered that the interactions between sleep disorders and LPRD mostly includes the following possible mechanisms: (1) Sleep disorders reduce the secretion of melatonin, promote the secretion of gastric acid/pepsin, increase the incidence of transient relaxation of lower esophageal sphincter, and causes corresponding gastrointestinal motility disorders and reflux diseases aymptoms by disrupting circadian rhythm; (2) Sleep disorders cause gastrointestinal motility disorders, thus increasing the incidence of reflux; (3) Sleep disorders stimulates the formation of excessive free radicals, inhibits the antioxidant capacity of cells, leading to or aggravates pathological damage of multiple systems, and finally induces various inflammation-related diseases; (4) Sleep disorder may also affect the reproduction of intestinal flora. The intestinal flora can regulate brain development by itself or metabolites, thus affecting physiological activities. The regulation of intestinal microecology may provide a new direction for the treatment of LPRD. (5) Sleep disorder is also associated with mental and psychological disorders (17). Lechien et al. (18) recognized that the risk of GERD was significantly and positively correlated with the severity of negative emotions, and a considerable part of the negative emotional effects were achieved through sleep disorders. This study showed that the HADS scores of patients with abnormal PSQI scores are lower than those of patients with normal PSQI scores, and there is a positive correlation between PSQI score and HADS scores. It is further confirmed that sleep disorder may be related to the occurrence of negative emotions in patients with LPRD patients. Improving sleep state is beneficial to reducing the patients' bad emotional state and improving the overall curative efficacy of LPRD.

In summary, sleep disorder may result in the appearance or aggravation of anxiety and depression in patients with LPRD, and sleep disorder is an independent risk factor for the development of LPRD. Clinical attention to the treatment of sleep disorders in patients with LPRD may be conducive to improving the efficacy of LPRD.

## Data Availability Statement

The original contributions presented in the study are included in the article/supplementary material, further inquiries can be directed to the corresponding author/s.

## Ethics Statement

The studies involving human participants were reviewed and approved by the Ethics Committee of the Second People's Hospital of Changzhou. The patients/participants provided their written informed consent to participate in this study.

## Author Contributions

YL was responsible for the writing and revision of the manuscript. JW was responsible for the design of the study. FX was responsible for the inclusion of cases. XG was responsible for the statistics of the results. LJ was the instructor of the entire study. All authors contributed to the article and approved the submitted version.

## Conflict of Interest

The authors declare that the research was conducted in the absence of any commercial or financial relationships that could be construed as a potential conflict of interest.

## Publisher's Note

All claims expressed in this article are solely those of the authors and do not necessarily represent those of their affiliated organizations, or those of the publisher, the editors and the reviewers. Any product that may be evaluated in this article, or claim that may be made by its manufacturer, is not guaranteed or endorsed by the publisher.
